# Transferrin Receptor-Targeted Iduronate-2-sulfatase Penetrates the Blood-Retinal Barrier and Improves Retinopathy in Mucopolysaccharidosis II Mice

**DOI:** 10.1021/acs.molpharmaceut.3c00736

**Published:** 2023-10-20

**Authors:** Atsushi Imakiire, Hideto Morimoto, Hidehiko Suzuki, Tomomi Masuda, Eiji Yoden, Asuka Inoue, Hiroki Morioka, Takashi Konaka, Ayaka Mori, Ryoji Shirasaka, Ryo Kato, Tohru Hirato, Hiroyuki Sonoda, Kohtaro Minami

**Affiliations:** Research Division, JCR Pharmaceuticals, 1-5-4 Murotani, Nishi-ku, Kobe 651-2241, Japan

**Keywords:** mucopolysaccharidosis II, retinopathy, blood-retinal barrier, transferrin receptor, electroretinography, pabinafusp alfa

## Abstract

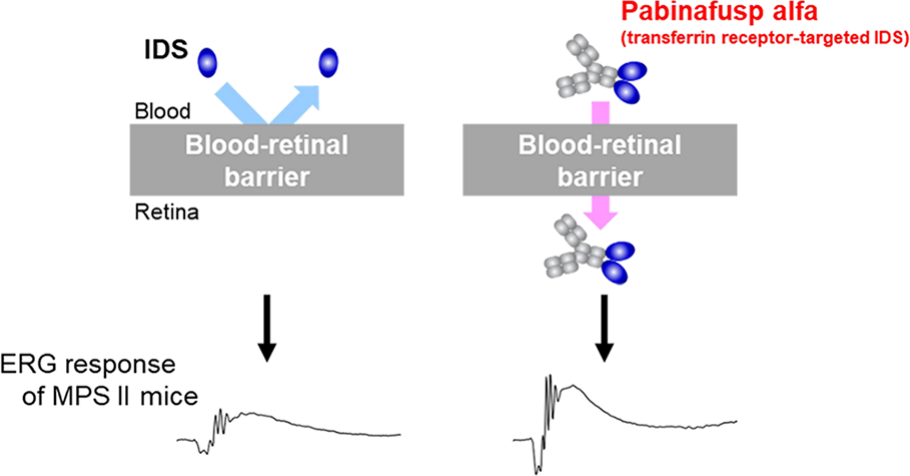

Mucopolysaccharidoses (MPSs) make up a group of lysosomal storage diseases characterized by the aberrant accumulation of glycosaminoglycans throughout the body. Patients with MPSs display various signs and symptoms, such as retinopathy, which is also observed in patients with MPS II. Unfortunately, retinal disorders in MPS II are resistant to conventional intravenous enzyme-replacement therapy because the blood-retinal barrier (BRB) impedes drug penetration. In this study, we show that a fusion protein, designated pabinafusp alfa, consisting of an antihuman transferrin receptor antibody and iduronate-2-sulfatase (IDS), crosses the BRB and reaches the retina in a murine model of MPS II. We found that retinal function, as assessed by electroretinography (ERG) in MPS II mice, deteriorated with age. Early intervention with repeated intravenous treatment of pabinafusp alfa decreased heparan sulfate deposition in the retina, optic nerve, and visual cortex, thus preserving or even improving the ERG response in MPS II mice. Histological analysis further revealed that pabinafusp alfa mitigated the loss of the photoreceptor layer observed in diseased mice. In contrast, recombinant nonfused IDS failed to reach the retina and hardly affected the retinal disease. These results support the hypothesis that transferrin receptor-targeted IDS can penetrate the BRB, thereby ameliorating retinal dysfunction in MPS II.

## Introduction

Mucopolysaccharidosis (MPS), a group of lysosomal storage disorders, includes several disease types based on which enzymes are deficient.^1^ Of these, MPS type II, also known as Hunter syndrome, is characterized by the accumulation of glycosaminoglycans (GAGs), heparan sulfate (HS), and dermatan sulfate (DS), which are substrates for iduronate-2-sulfatase (IDS), throughout the body.^1−4^ The deposition of these substrates in the lysosome impairs cell functions in various tissues, leading to a wide spectrum of clinical manifestations, such as a coarse face, hepatosplenomegaly, cardiac valve disease, respiratory failure, musculoskeletal abnormalities, and vision loss. Patients with severe forms of MPS II also progressively develop neuronopathy with multiple central nervous system (CNS) symptoms.^1−4^ Although enzyme-replacement therapy (ERT) is available for the treatment of patients with MPS II,^5^ conventional intravenous ERT with recombinant IDS is not effective against CNS symptoms or retinopathy.^6^

The blood-brain barrier (BBB) consists of brain capillary endothelial cells with tight junctions surrounded by pericytes and astrocytes.^7^ Although the BBB limits the transport of macromolecules between the blood and brain, certain endogenous peptides and proteins are capable of crossing via specific receptors expressed on the endothelial cell surface by receptor-mediated transcytosis.^8^ By exploiting this endogenous transport system, the delivery of intravenously injected protein drugs to the brain has been previously examined.^9−21^ Pabinafusp alfa, a BBB-penetrating IDS-targeted transferrin receptor (TfR) generated by fusion with an antihuman TfR antibody, was successfully delivered to neurons in the brains of cynomolgus monkeys and MPS II model mice. It preserved neurological function in mice as determined by several experimental tests.^18,22,23^ Pabinafusp alfa has been evaluated in clinical studies and is currently available for the treatment of patients with all types of MPS II in Japan, including the neuronopathic type.^24−26^

The retina is protected from systemic circulation by two distinct blood-retinal barriers (BRB). The outer BRB is formed by retinal pigment epithelial (RPE) cells, which separate the outer surface of the retina from the choriocapillaris. The inner BRB is structurally similar to the BBB and consists of retinal capillary endothelial cells with tight junctions surrounded by pericytes and Müller’s glial cells.^27^ Nourishment of the human retina is largely attributed to retinal capillaries via the inner BRB.^28,29^ The iron transport system in the retina is also similar to that of the brain. Both RPE cells (outer BRB) and retinal capillary endothelial cells (inner BRB) express TfR, which imports the transferrin-bound iron into the retina.^30,31^ These findings prompted us to investigate whether the BBB-penetrating IDS could also traverse the BRB to reach the retina and affect visual function. In the present study, we demonstrate the retinal delivery and efficacy of pabinafusp alfa following intravenous administration in an MPS II model.

## Materials and Methods

### Test Substances

Pabinafusp alfa (JR-141) was generated as described previously.^18^ Recombinant nonfused human IDS was produced in-house in Chinese hamster ovary cells cultured in serum-free medium similar to pabinafusp alfa. The calculated molecular weights (without sugar chains) of pabinafusp alfa and the nonfused IDS were 265,110.93 and 59,274.99, respectively. The purified recombinant proteins were stored at temperatures below −70 °C until use.

### Animals

The MPS II model animals were *Ids* deficient (*Ids*-KO) mice with a C57BL/6 background.^32^ Human TfR-expressing *Ids*-deficient (hTfR-KI/*Ids*-KO) mice were also used as MPS II model animals and produced by crossbreeding *Ids*-KO mice with hTfR-KI mice.^18^ C57BL/6 mice were purchased from Charles River, Japan (Yokohama, Japan). All animal experiments were performed in accordance with the ARRIVE guidelines 2.0, and the protocols (JR141-P2202, P2203, and PK2301) were approved by the Animal Care and Use Committee of JCR Pharmaceuticals. Mice were housed under a 12-h light-dark cycle with free access to water and a standard rodent chow diet.

### Retinal Drug Delivery

Distribution of the test substances to the retina of MPS II mice was determined by immunohistochemistry after a single intravenous dose of pabinafusp alfa or nonfused IDS at 2 mg/kg body weight. Mice were euthanized under anesthesia 17 h after the injection, and their eyes were removed and embedded in the OCT compound. The protocol for immunostaining using anti-hIDS antibody was described previously with some modifications.^18,23^ Briefly, frozen sections were fixed with 4% paraformaldehyde and blocked with streptavidin/biotin blocking solution (Vector Laboratories, Newark, CA) and superblock (Thermo Fisher Scientific, Waltham, MA). The sections were incubated with anti-hIDS antibody at 4 °C and then with antirabbit IgG H&L HRP-polymer (Abcam, Cambridge, UK). The specimens were reacted with tyramide-biotin amplification reagent (Thermo Fisher Scientific) and then with streptavidin-peroxidase polymer (Sigma-Aldrich, St. Louis, MO). Signals were visualized with ImmPACT NovaRED (Vector Laboratories). Counterstaining was done by hematoxylin. We also quantified the IDS enzyme in the retina by an electrochemiluminescence-based immunoassay.^18^ Isolation of the retina was performed, as described below.

### Long-Term Efficacy Study

#### Study Design

To evaluate the long-term effects on the retina, we administered pabinafusp alfa to male MPS II (hTfR-KI/*Ids*-KO) mice intravenously at 0.5 or 2 mg/kg body weight through the tail vein once a week for 40 weeks, beginning from the age of 10 weeks. Nonfused IDS at 0.5 mg/kg body weight (the approved dosage of clinically available IDS, idursulfase^33^) or physiological saline was administered to the mice in the same manner. hTfR-KI mice were designated the nonpathological control (normal control). Based on previous studies,^18,22,23^ 14 animals were allocated to each group. The number of animals for each assessment is indicated in the corresponding figure. The body weight of the mice was measured before each administration of drug. Because infusion-associated reactions were not severe, no immunosuppressant was used in this study.

#### Electroretinography (ERG)

Scotopic ERG was recorded bilaterally before (baseline) and 38 weeks after the repeated administration of the test substances. The day before ERG monitoring, the mice were dark-adapted overnight in a dark room. All of the subsequent procedures were performed under deep-red light to preserve the dark adaptation. The animals were anesthetized with three types of mixed anesthetic agents (medetomidine/midazolam/butorphanol: 0.75/4.0/5.0 mg/kg). After mydriasis with 1% atropine sulfate, the mice were placed on a heating pad in a Ganzfelt dome (Mayo, Aichi, Japan). The reference electrode was inserted into the mouth, and the ground electrode was attached to the tail. The contact lens electrodes were placed on the center of the cornea, and the ERG response was recorded using the PuRec recording system (Mayo). For recording the scotopic responses, single white-flash stimuli at 0.00001, 0.0001, 0.001, 0.01, 0.1, 1, and 10 cd s/cm^2^ were given. The results were averaged for the left and right eyes. In general, ERG testing was performed according to the International Society for Clinical Electrophysiology of Vision Standard.^34^

#### Tissue Collection and HS Measurement

One week after the last (40th) dose, the mice were euthanized under anesthesia. The brain (visual cortex) and ocular tissues were collected and processed to measure the HS concentration. To isolate the retina and optic nerve, the right eyeballs were enucleated and placed in a Petri dish filled with cold PBS. The extra tissues surrounding the eyeballs were removed with microdissection scissors under a stereomicroscope. The optic nerve was cut from each eyeball. Then, an incision was made along the ora serrata through a hole introduced by a needle, and the lens and cornea-iris were removed. Finally, the retina was isolated from the RPE/choroid/sclera complex.^35^ The isolated tissues were stored below −80 °C until use. HS measurement was performed as previously described.^36^

#### Histological Analyses

The mice were humanely euthanized through exsanguination from the abdominal aorta under anesthesia. Subsequently, the entire body underwent perfusion with physiological saline. The collected eyeballs were then subjected to immersion fixation in 10% neutral-buffered formalin. These fixed eyeballs were subsequently embedded in paraffin and sliced into 4 μm sections. Standard protocols, as previously outlined,^22,23^ were followed for H&E-staining.

#### Retinal Cell Isolation and Cell Count

To isolate single cells from the retina, the tissue was cut into small pieces (1–2 mm) using scissors and incubated for 30 min at 37 °C in PBS containing 2% fetal bovine serum and 1 mg/mL collagenase I (Wako Chemicals, Richmond, VA). The suspended cells were filtered using a 100-μm cell strainer and stained with Muse Count & Viability Reagent (Luminex, Austin, TX). Cell viability and total cell number were determined using the Guava Muse Cell Analyzer (Luminex).

### Statistics

Data are presented as the mean ± SEM. Statistical analysis was conducted using KyPlot 6.0 statistics software (KyensLab). We utilized an unpaired *t* test to compare differences between the normal control (WT) and disease control (KO) groups and a Tukey-Kramer test to compare differences between treatment groups. Statistical significance was set at *P* < 0.05.

## Results

### Retinal Dysfunction in MPS II Mice

**Figure 1:**
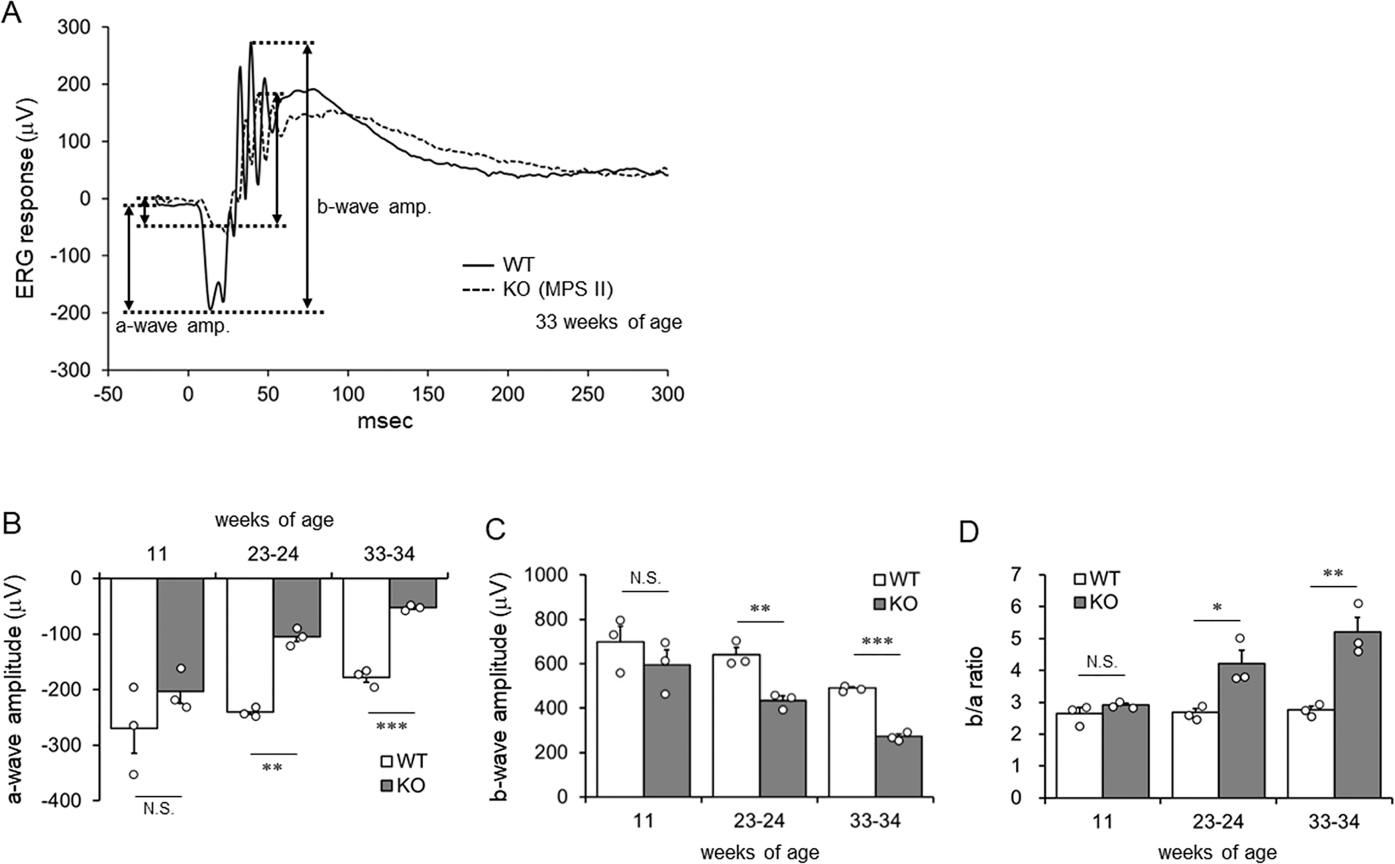
ERG responses of WT and MPS II mice at different ages. (A) Raw ERG traces from representative WT and *Ids*-KO (MPS II) mice at 33 weeks of age. Scotopic ERG responses were recorded at 10 cd s/cm^2^. Amplitudes of a-wave and b-wave were indicated by double arrows. (B–D) Amplitudes of photoreceptor-generated a-wave (B), amacrine/bipolar cell-generated b-wave (C), and *b*/*a* ratio (D). Bars indicate the mean with SEM (*n* = 3). Open circles indicate values for individual mice. *P < 0.05, **P < 0.01, and ***P < 0.001 (WT vs KO, unpaired *t* test). N.S., not significant; WT, wild-type mice; and KO, *Ids*-KO (MPS II) mice.

Retinal function in MPS II (*Ids*-KO) mice with different ages was evaluated by ERG. The ERG was recoded at 10 cd s/cm^2^, which includes both cone and rod responses (Figure 1A). We found that both a-wave and b-wave amplitudes of ERG tended to be decreased in MPS II mice as early as 11 weeks of age. The trend became clear and statistically significant with age (Figure 1B,C). Furthermore, the ratio of b-wave to a-wave increased in older MPS II mice compared to WT mice of the same age (Figure 1D), indicating that the impact of MPS II disease was greater on the photoreceptor-generated a-wave than on the amacrine/bipolar cell-generated b-wave.

### Retinal Delivery of Pabinafusp Alfa

**Figure 2:**
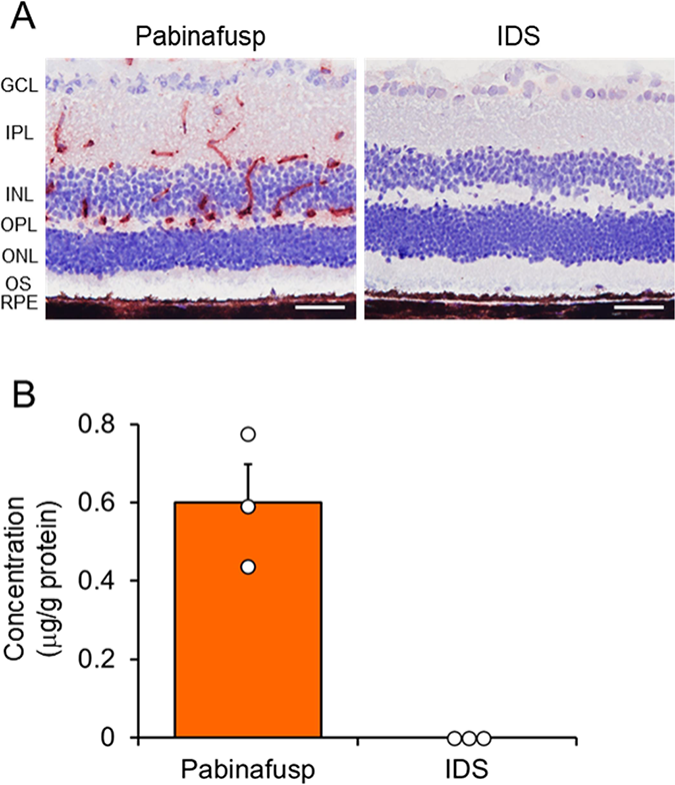
Retinal delivery of pabinafusp alfa. (A) Immunostaining of the human IDS enzyme in the retina after intravenous administration of 2 mg/kg of pabinafusp alfa or nonfused IDS to MPS II mice. Red-brown signals indicate positive staining for IDS. Counterstaining was performed by hematoxylin. Scale bars: 50 μm. (B) Quantification of IDS enzyme concentration by electrochemiluminescence. Bars indicate mean with SEM (*n* = 3). Open circles indicate values for individual mice. Retinas were isolated 17 h after administration. Pabinafusp, pabinafusp alfa; IDS, nonfused recombinant human IDS; GCL, ganglion cell layer; IPL, inner plexiform layer; INL, inner nuclear layer; OPL, outer plexiform layer; ONL, outer nuclear layer; OS, outer segment; and RPE, retinal pigment epithelium.

We examined whether pabinafusp alfa could deliver the IDS enzyme to the retina following a single intravenous administration of 2 mg/kg in MPS II mice (hTfR-KI/*Ids*-KO). Immunostaining for human IDS revealed that the enzyme was not present in the retina upon intravenous injection of nonfused recombinant human IDS (Figure 2A). This aligns with the notion that the BRB limits the delivery of macromolecules from the circulation.^37^ In contrast, the IDS enzyme was detected in the retina following intravenous administration of pabinafusp alfa (Figure 2A). Consistent with these findings, quantification of the enzyme concentration through an electrochemiluminescence-based immunoassay revealed that the IDS enzyme was measurable in the retina exclusively after pabinafusp alfa injection (Figure 2B). These results affirm that the TfR-targeted fusion protein pabinafusp alfa facilitates the delivery of IDS enzyme to the retina by crossing the BRB.

### HS Reduction by Pabinafusp Alfa

**Figure 3:**
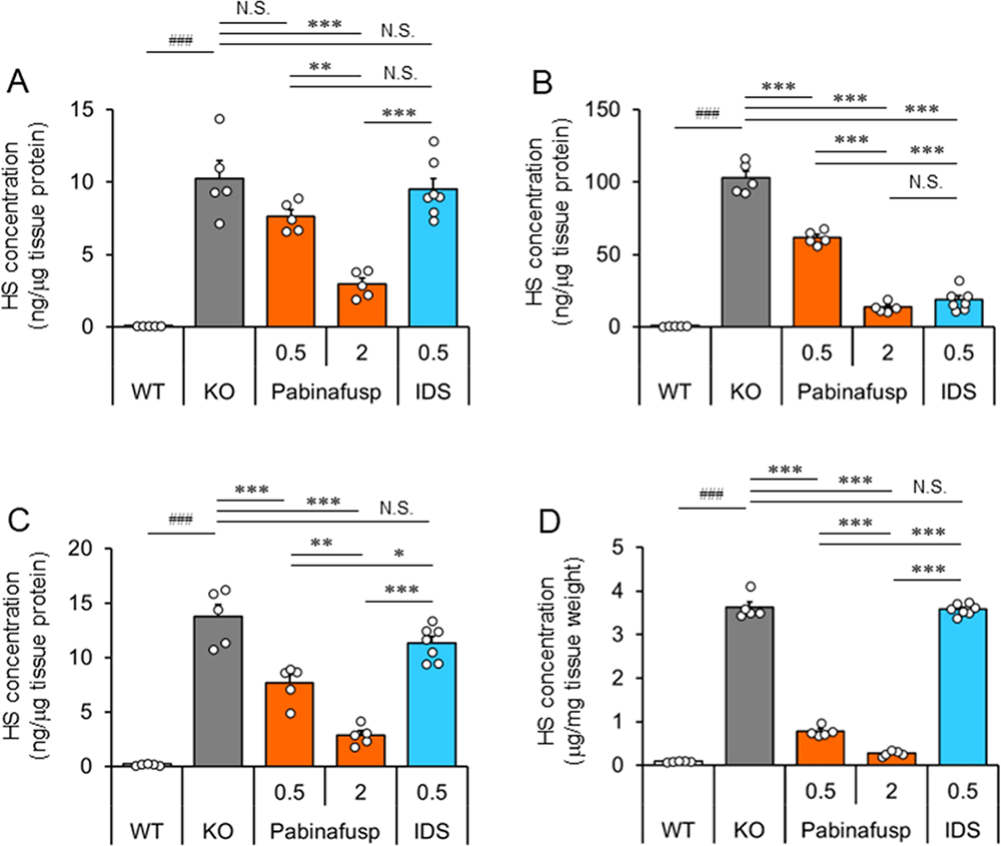
Effect of pabinafusp alfa on the HS concentration in tissues involved in visual function. HS concentration in the retina (A), RPE/choroid/sclera complex (B), optic nerve (C), and visual cortex of the brain (D). HS concentration was measured 1 week after the final (40th) dose administration. Bars indicate the mean with SEM (*n* = 5–7). Open circles indicate values for individual mice. The numbers on the *X*-axis indicate the dose in mg/kg. ^###^*P* < 0.001 (WT vs KO, unpaired *t* test), *P < 0.05, **P < 0.01, and ***P < 0.001 (between treatment groups, Tukey-Kramer test). N.S., not significant; WT, wild-type mice; KO, vehicle-treated hTfR-KI/*Ids*-KO (MPS II) mice; Pabinafusp, pabinafusp alfa; and IDS, nonfused recombinant human IDS.

We examined whether pabinafusp alfa reduces the HS concentration in a 40-week repeated dose study using MPS II (KO) mice. In MPS II mice, HS concentration was markedly increased in the retina, RPE/choroid/sclera complex, optic nerve, and visual cortex of the brain (Figure 3A–D), all of which are involved in visual function. However, the effect of nonfused IDS was limed to decreasing HS concentration only in the RPE/choroid/sclera complex, which primarily consists of tissues located outside the BRB. In contrast, pabinafusp alfa reduced the concentration in all these tissues in a dose-related manner (Figure 3A–D).

### Effect of Pabinafusp Alfa on Retinal Function Assessed by ERG

**Figure 4:**
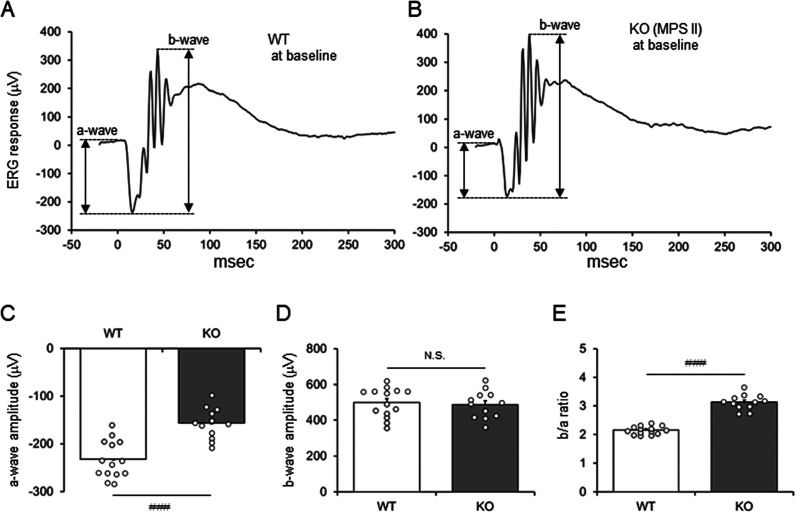
Baseline measurement of ERG before the initiation of treatment. (A,B) ERG responses from representative WT (A) and KO (MPS II, B) mice at 10 cd s/cm^2^. Raw ERG traces are shown. Double arrows indicate a-wave and b-wave amplitudes. (C–E) Amplitude of a-wave (C), b-wave (D), and b/a ratio (E) of the ERG. Data are from mice before initiation of the treatment (baseline). Bars indicate the mean with SEM (*n* = 14 for WT and 12 for KO). Open circles indicate values for individual mice. ^###^*P* < 0.001 (WT vs KO, unpaired *t* test), N.S., not significant; WT, wild-type mice; KO, vehicle-treated hTfR-KI/Ids-KO (MPS II) mice; Pabinafusp, pabinafusp alfa; and IDS, nonfused recombinant human IDS.

**Figure 5:**
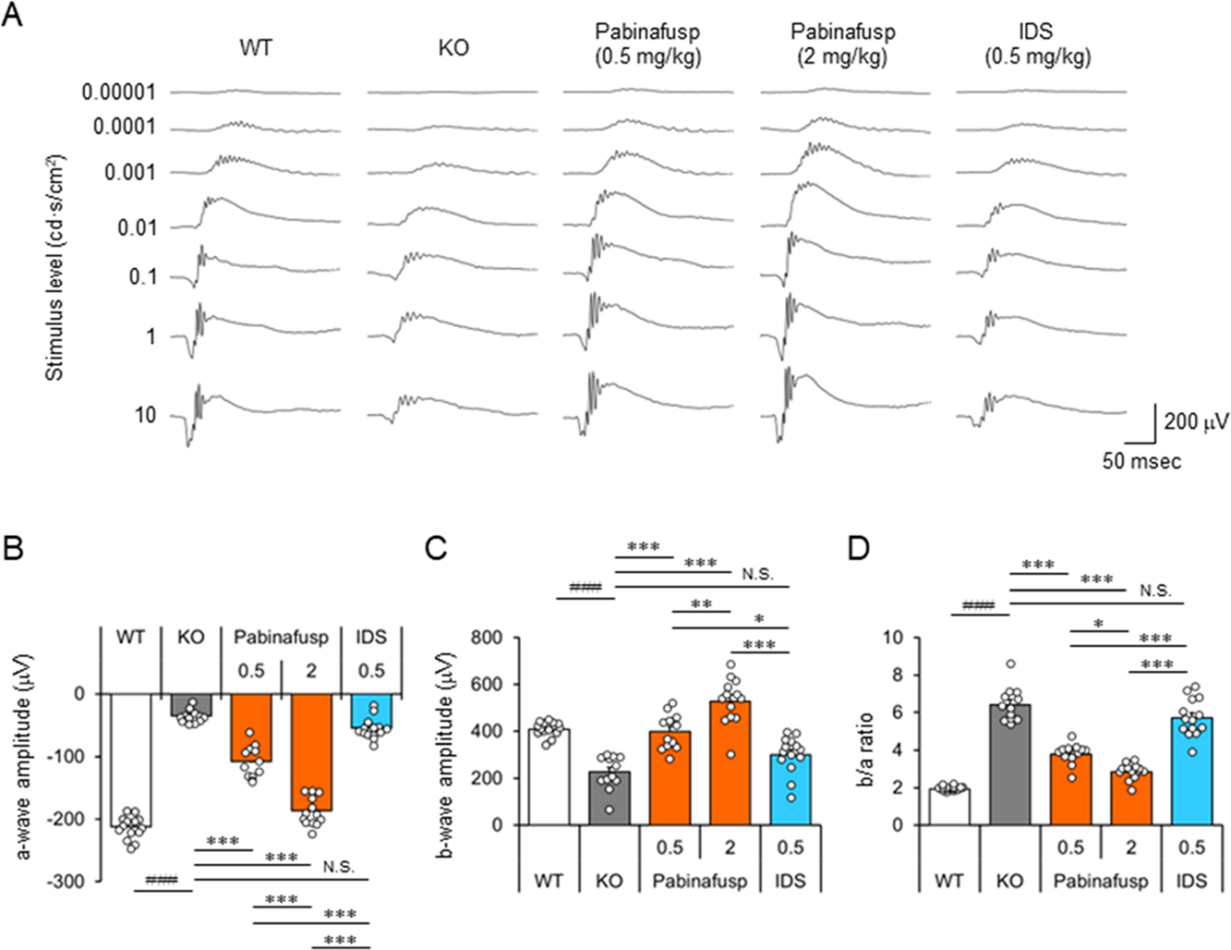
Effect of a chronic treatment with pabinafusp alfa on retinal function. (A) ERG responses of representative mice resulting from different intensity stimuli after a 38-week treatment. (B–D) Amplitude of a-wave (B), b-wave (C), and b/a ratio (D) of ERG at 10 cd s/cm^2^ after treatment. Bars indicate the mean with SEM (*n* = 12–14). Open circles indicate values for individual mice. The numbers on the *X*-axis indicate the dose in mg/kg. ^###^*P* < 0.001 (WT vs KO, unpaired *t* test), *P < 0.05, **P < 0.01, and ***P < 0.001 (between treatment groups, Tukey-Kramer test). N.S., not significant; WT, wild-type mice; KO, vehicle-treated hTfR-KI/*Ids*-KO (MPS II) mice; Pabinafusp, pabinafusp alfa; and IDS, nonfused recombinant human IDS.

We examined the effect of prolonged treatment with pabinafusp alfa on retinal function as assessed by ERG. At the baseline (10 weeks of age), MPS II (KO) mice exhibited a decreased a-wave but a normal b-wave of ERG response at 10 cd s/cm^2^ compared to that of WT mice (Figure 4). This indicated the presence of a photoreceptor function defect and normal amacrine/bipolar cell function at the beginning of treatment. After 38 weeks of repeated dosing, the threshold light stimulus necessary to elicit ERG responses was increased (indicating impairment) in vehicle-treated MPS II mice compared with WT mice (Figure 5A). Moreover, both the a-wave and b-wave of the ERG response were profoundly reduced in vehicle-treated MPS II mice (Figure 5B,C), suggesting the onset of retinopathy. While treatment with nonfused IDS had either no effect or a limited effect on the ERG response in MPS II mice, mice treated with pabinafusp alfa showed ERG responses similar to those of WT mice (Figure 5A). Specifically, at the approved clinical dose of 2 mg/kg, the amplitudes of both the a-wave and b-wave (including their ratio) were close to those of WT levels (Figure 5B–D). Notably, the photoreceptor-generated a-wave amplitude exhibited some improvement from the baseline (Figure S1) and nearly normalized to WT levels (Figure 5B) following treatment with pabinafusp alfa at 2 mg/kg.

### Histological Analyses of the Retina after the Treatment

**Figure 6:**
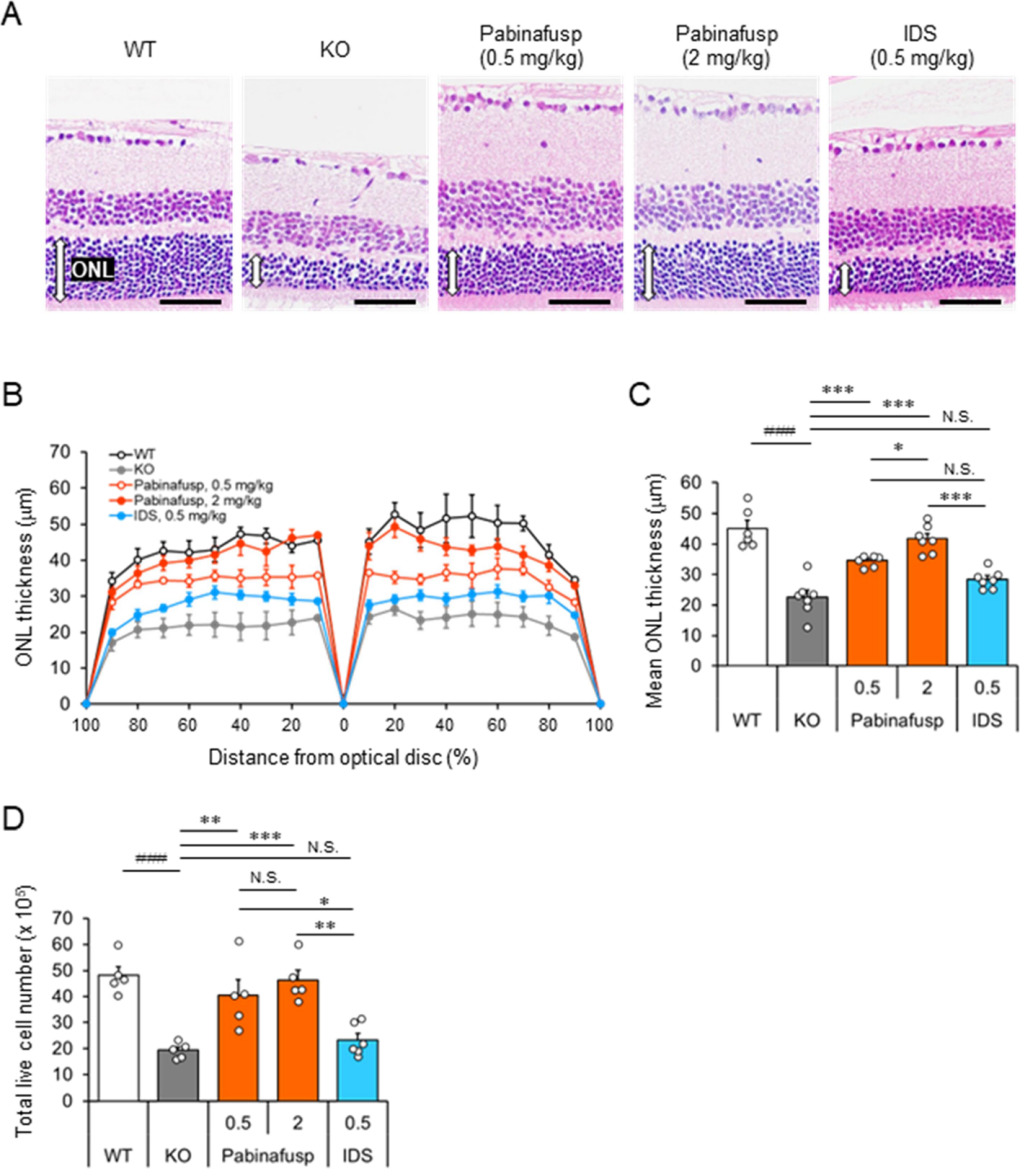
Histological analyses of the retina after the treatment. (A) H&E-staining of the retina. White double arrows indicate the thickness of the outer nuclear layer (ONL). Scale bars, 50 μm. (B) Thickness of the ONL at 18 positions on both sides of the retina. Values are the means ± SEM (*n* = 6–7). (C) Mean ONL thickness. Bars indicate the mean with SEM (*n* = 6–7). (D) Total cell number in the retina. Bars indicate the mean with SEM (*n* = 5). Open circles indicate values for individual mice (C and D). ^###^*P* < 0.001 (WT vs KO, unpaired *t* test), *P < 0.05, **P < 0.01, and ***P < 0.001 (between treatment groups, Tukey-Kramer test). N.S., not significant; WT, wild-type mice; KO, vehicle-treated hTfR-KI/*Ids*-KO (MPS II) mice; Pabinafusp, pabinafusp alfa; and IDS, nonfused recombinant human IDS.

To investigate the underlying mechanisms responsible for the effects of pabinafusp alfa on retinal function, we performed histological analyses of the retina following treatment. In the MPS II mouse retina, no apparent changes were noted in the inner nuclear layer (INL), which includes amacrine and bipolar cells (Figure 6A). However, there was a significant reduction in the thickness of the outer nuclear layer (ONL), which contains photoreceptors (Figure 6A–C). While the ONL of nonfused IDS-treated MPS II mice exhibited only a slight increase in thickness compared to vehicle-treated MPS II mice, those treated with pabinafusp alfa at 2 mg/kg demonstrated a similar ONL thickness to WT mice (Figure 6A–C). Furthermore, the total live cell number in the retina decreased in vehicle-treated MPS II mice, whereas pabinafusp alfa, but not nonfused IDS, significantly suppressed the loss of retinal cells (Figure 6D). These results imply that a substantial loss of photoreceptors (rod and/or cone cells) occurs in MPS II mice, a loss that can be prevented through treatment with pabinafusp alfa.

## Discussion

Vision loss is a significant complication associated with MPSs^38−41^ and has a negative impact on the independence and quality-of-life of patients. Although corneal clouding is the most frequently observed ocular problem in MPSs, it is not as common in MPS II.^42−44^ In contrast, a considerable proportion of patients with MPS II develops retinopathy, probably due to GAG deposition in the RPE and interphotoreceptor matrix, which can lead to progressive loss of photoreceptor layers.^44−46^ Optical coherence tomography analysis has demonstrated retinal degeneration in MPS II.^45,47−49^ Our murine model of MPS II (hTfR-KI/*Ids*-KO) recapitulated eye defects, including retinal degeneration (cell loss) and decreased ERG responses, mirroring those observed in patients with MPS II.^50,51^

Conventional intravenous ERT fails to address retinal complications in MPS II because of the BRB, which limits the transport of macromolecules from the systemic circulation to the retina.^37^ Pabinafusp alfa, a fusion protein consisting of antihuman TfR antibody and IDS, was designed to penetrate the BBB into the brain by targeting TfR.^18^ Because the structure and iron transport mechanism of the BRB closely resemble those of the BBB,^27,30,31^ we hypothesized that the TfR-targeting strategy could also be applied to retinal drug delivery. This was confirmed in the present study by detecting the IDS enzyme in the retina following the intravenous administration of pabinafusp alfa in MPS II mice.

Our data show that substantial HS deposition was observed not only in the retina but also in the optic nerve and visual cortex, suggesting that pathological changes occur in these tissues and the overall visual system is impaired in MPS II mice. In the present study, we focused on the retina and found that the total cell number in the retina was markedly decreased in the MPS II mice. Additionally, histological analysis revealed a notable decrease in the ONL thickness, indicating a loss of photoreceptor cells. Because the largest proportion of mouse retina is occupied by rod cells,^52^ rod cell loss should be a major cause of the decreased ONL thickness and total cell number in the retina of MPS II mice. In this context, early intervention with pabinafusp alfa almost completely suppressed the development of these pathological changes, thereby preserving visual function, as assessed by ERG in MPS II mice. Importantly, we noted that the photoreceptor-generated a-wave amplitude of ERG was already impaired in young MPS II mice at the beginning of treatment (10 weeks of age). However, chronic treatment with pabinafusp alfa improved and normalized the a-wave amplitude (Figures 4 and 5). These results suggest that the defect in photoreceptor function in young MPS II mice is reversible and that pabinafusp alfa can restore the function. Nonetheless, it remains an open question whether similar results can be achieved in human MPS II patients.

We observed that nonfused IDS had a modest impact on the ERG response in MPS II mice, albite without statistical significance (Figure 5). Additionally, there was a reduction in HS deposition in the RPE/choroid/sclera complex following nonfused IDS treatment (Figure 3). This suggests a possibility that a certain amount of IDS may have entered the RPE cells, which constitute the outer BRB and provide support to the retina. This could have resulted in a reduction in the HS in these cells, potentially suppressing the loss of RPE function in MPS II mice. Give that the crucial role of the RPE in supporting photoreceptor survival and function,^53,54^ maintaining RPE function to some extent might indirectly mediate the effects of nonfused IDS on photoreceptor function and ERG responses. Indeed, the ONL exhibited a slightly greater thickness in IDS-treated mice compared with vehicle-treated mice (Figure 6A–C). Notably, the TfR-targeted IDS pabinafusp alfa has the capability to directly access retinal cells within the BRB, including photoreceptors. This likely contributes to the nearly complete suppression of retinopathy development.

A limitation of this study is the lack of direct evidence to elucidate the mechanistic aspects of impaired retinal function in MPS II mice. While HS deposition could potentially trigger pathologic changes in the retina, leading to decreased ERG responses, it remains unclear which specific cell types primarily contribute to this impairment. It is possible that HS deposition directly damages photoreceptor cells, particularly rod cells, while the loss or dysfunction of RPE cells may indirectly impact photoreceptors, as indicated by studies conducted in MPS IIIA/B mice.^55,56^ Further studies are warranted to address these aspects comprehensively.

In conclusion, we demonstrated that MPS II mice progressively developed retinopathy. HS deposition was observed in the retina of the mice, which likely resulted in retinal cell damage. Importantly, we showed that TfR-targeted IDS pabinafusp alfa effectively reached the retina by crossing the BRB following intravenous administration in MPS II mice. Pabinafusp alfa reduced HS levels within the retina of the mice and preserved or even improved retinal function when treatment was begun at an early age. These results shed light on the management of challenging eye diseases in MPS II patients.

## Supporting Information Available

Changes in the ERG responses before (baseline) and after (end point) treatment (PDF)

## Notes

A.I., H.M., H.S., T.M., E.Y., A.I., H.M., T.K., A.M., R.S. R.K., T.H., H.S., and K.M. are employees and/or stockholders of JCR Pharmaceuticals Co., Ltd.

## Abbreviations

BBB: blood-brain barrier
BRB: blood-retinal barrier
CNS: central nervous system
DS: dermatan sulfate
ERG: electroretinography
ERT: enzyme-replacement therapy
GAG: glycosaminoglycan
HS: heparan sulfate
IDS: iduronate-2-sulfatase
KO: knockout disease control
MPS: mucopolysaccharidosis
TfR: transferrin receptor
WT: wild-type normal control
