# Supplementary material for: Transferrin Receptor-Targeted Iduronate-2-sulfatase Penetrates the Blood-Retinal Barrier and Improves Retinopathy in Mucopolysaccharidosis II Mice
Source: Mol Pharm. 2023 Oct 20;20(11):5901–9. doi: 10.1021/acs.molpharmaceut.3c00736 (PMC10630942; doi:10.1021/acs.molpharmaceut.3c00736)
Supplement: Supplementary file 1 — mp3c00736_si_001.pdf [file mp3c00736_si_001.pdf]

Supporting Information

Transferrin receptor-targeted iduronate-2-sulfatase penetrates the blood-retinal barrier and improves retinopathy in mucopolysaccharidosis II mice

Atsushi Imakiire, Hideto Morimoto, Hidehiko Suzuki, Tomomi Masuda, Eiji Yoden, Asuka Inoue, Hiroki Morioka, Takashi Konaka, Ayaka Mori, Ryoji Shirasaka, Ryo Kato, Tohru Hirato, Hiroyuki Sonoda, Kohtaro Minami

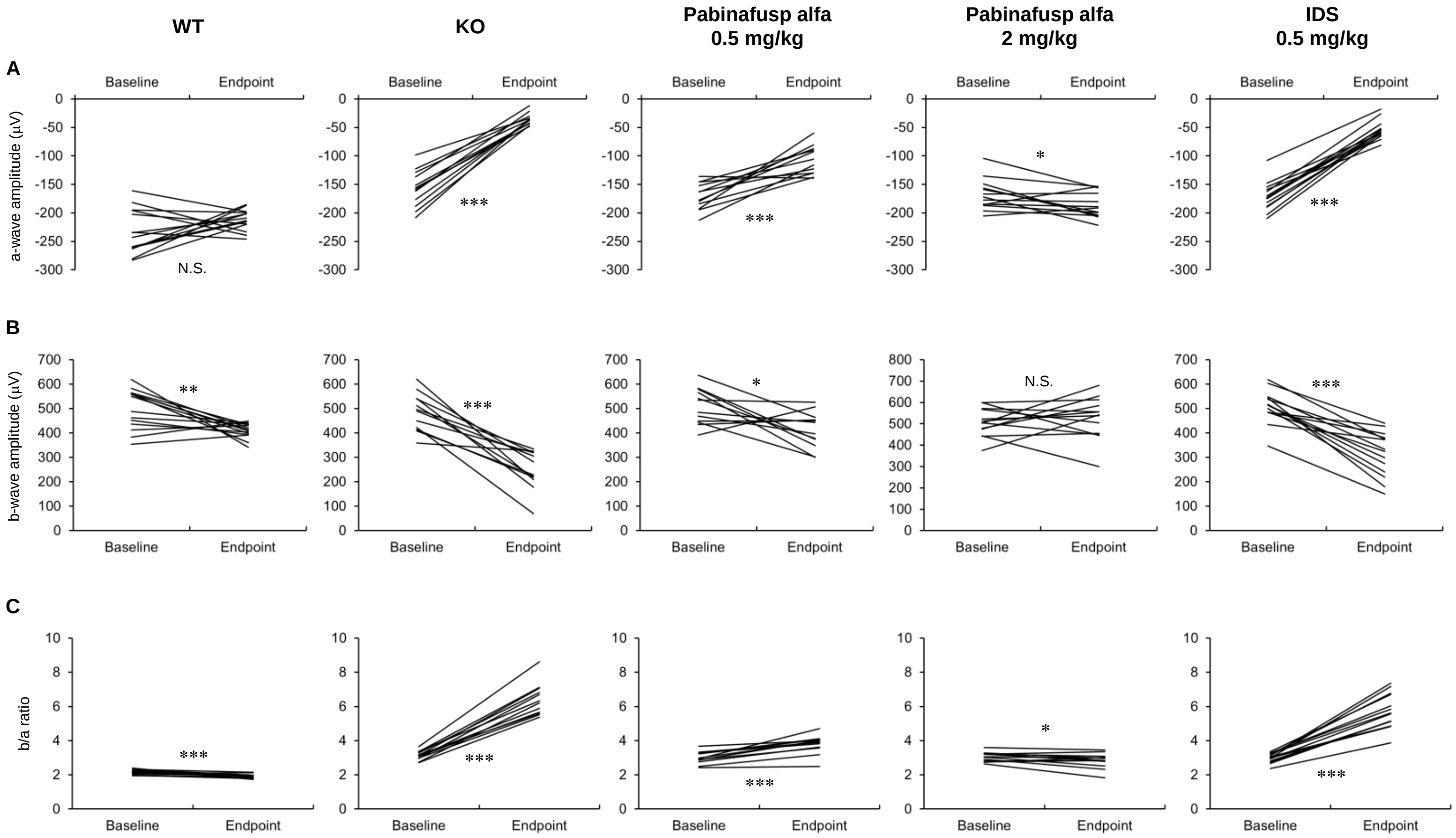

Figure S1. Changes in the ERG responses before (baseline) and after (endpoint) treatment. (A) a-wave amplitude. (B) b-wave amplitude. (C) b/a ratio. Each straight line represents the change in the value of an individual animal. *P < 0.05, **P < 0.01, ***P < 0.001 (paired t-test between baseline and endpoint). N.S., not significant; WT, wild-type mice; KO, vehicle-treated hTfR-KI/Ids-KO (MPS II) mice; IDS, non-fused recombinant human IDS.
